# Forceps Delivery

**Published:** 1889-04

**Authors:** W. D. Greene

**Affiliations:** 444 Elk Street; Buffalo, N. Y.


### *FORCEPS DELIVERY.*[1]

By W. D. GREENE, M. D., Buffalo, N. Y.

Up to the middle of the seventeenth century, nearly all obstetric practice was in the hands of midwives. The physician was called to treat the ordinary ills of life, but in the purely physiological act of childbirth, the midwife alone officiated. The physician must have been occasionally consulted, however, for it seems he recognized the necessity of some instrument to assist nature in the delivery of the child in difficult labors. And such an instrnment was invented—the forceps. It was invented by the Chamberlens, of England, and kept in secrecy for half a cen-

1. Read before the Buffalo Obstetrical Society.

tury. They were not generally known until about 1753, when they were first brought to the notice of the profession at large. They had their friends and enemies, Lamotte condemning them in strong terms. From the Chamberlen forceps to the classic forceps of to-day is a long jump, but in the latter we have, undoubtedly, the instrument in its perfection—an instrument which is praised by all conversant with its use—one which saves more lives than any other appliance connected with our art. It is not my intention, in this paper, to describe the different forceps and their modes of application, but shall be content if any light can be thrown upon the subject of how often, and under what circumstances they should be used. When can we safely use forceps without injury, but rather with benefit to the mother and her offspring? is a question every intelligent physician often propounds to himself. As the forceps of Tarnier have been so much praised, and also so thoroughly condemned, by different members of the medical profession, a passing notice might be profitable. Medical opinion is largely divided as to their superiority over the classic forceps. It has had several modifications, and still there are practitioners who believe the first model was the best. Pajot says of it:

"A complicated instrument, deprived of all lever property, less easy to introduce and manage, only able to do what all other forceps may; an instrument, still, of improved value where the head is movable above the superior strait; fenestræ, useful where delivery may be accomplished with dressing forceps, but useless otherwise on account of their small size; an instrument said to be of value because by it traction may be made in the pelvic axis—a superfluous advantage in ninety cases out of 100, and still to be proved in the remaining ten."

Charpentier points out one advantage it possesses over other forceps—that less force has to be made than with classic forceps. The same is not true at the superior strait, for there is the same difficulty with Tarnier's instrument as with the ordinary one, in carrying it sufficiently backwards, and the force to be used is identical in both instances. His objections are that a two-fold manipulation is requisite—one for the articulation of the handles, as in the classic forceps, the other for the insertion of the traction-rods:

"In order that these rods may functionate properly, they must be absolutely parallel; that is to say, the head must be seized symmetrically, and this, while easy enough in the cavity or at the inferior strait, is difficult if not impossible at the superior strait, the greatest objection being as follows: With classic forceps the operator is at all times conscious of the progress made by the fetal head. Using Tarnier's, however, this condition does not exist. When the head resists, greater traction is made. If it descends, all the better. If it resists, we pull harder still. Thus, nothing indicates the limit. Traction, therefore, may readily be exaggerated and serious damage done. Although the Tarnier forceps is just as firm in hold as the classic, it is, nevertheless, the compression-screw alone which retains the handles in apposition. The head may be seized incompletely with this instrument, even as with the classic, and consequently it is just as likely to slip. Now, with the ordinary forceps held in the hand, we know at once when it is slipping, and this may be prevented. Not so with Tarnier's. The hand pulls on the transverse bar of the traction-rods, and is no longer conscious of a slipping of the instrument from the head, which may result in vaginal and vulva lesions, not to speak of uterine—the greater, the stronger the traction exerted. When they are applied at or above the brim, since the perineal curve is absent, the disadvantages are as great in the classic forceps, and rotation occurs less readily."

Charpentier also says that, notwithstanding the assertion of its greatest advocates, it does endanger the integrity of the perineum as much as the classic forceps." Every invention, of whatever nature, must undergo a certain amount of adverse criticism, and the forceps of Tarnier is no exception. These criticisms have been severe, but have originated mostly from the rivals and confrères of Tarnier, which causes them to lose much of their force.* In the light of recent investigations, and the past experiences in using it, it would seem that it is more difficult of application at the brim of the pelvis than the classic forceps; but when applied in this situation, it is superior to the classic, from the fact that less traction has to be made, because the force can be more readily applied in the axis. The tendency to slip is also a matter to be considered, and the utmost watchfulness is needed.

Let us now turn to the consideration of what are the indications for the use of forceps of any description. Their use would seem to be indicated whenever the conditions exist which admit of their being used, and any danger menaces the mother or child. What I mean by the conditions are, that we should have a dilated or satisfactorily dilatable os, the membranes ruptured, and the head presenting. The preponderance of medical opinion seems to be that it is not applicable in pelvic presentations, while others hold contrary views, thus giving the forceps a wider field of usefulness. Granted, then, that it is indicated in certain pelvic presentations, and also in occasional occiput-posterior presentations, when labor is long-delayed or impossible, owing to feebleness or entire absence of expulsive pains; in great resistance of soft parts, contracted pelvis, large head, or face presentations. Whenever any of the common accidents occur, such as puerperal convulsions, hemorrhage, feebleness of heart's action, syncope, short cord, absence of rotation, etc.—anything, in short, which endangers the safety of mother or child—renders the use of the forceps imperative. The arrest of the head at or above the superior strait, and rigid and unyielding perineum, may also be classed under the same head. Supposing, then, in a given case, the os is dilated, the membranes have ruptured, the pelvis is not contracted, and still labor is not terminated, even where strong uterine contractions are present, how long are we to wait before resorting to the forceps? Just here there is a wide difference of opinion. Playfair says: "The use of the forceps is the rule, in place of the exception. They diminish, in a marked degree, infant mortality. No danger to mother, but often great danger from delay in their use." Leishman says: "Ergot, to increase the activity of the pains, is never perfectly safe, and the forceps should be preferred." Up to the time of Smellie, it was always taught that, with the head above the brim, version should be resorted to; but, since that time, the forceps is employed instead, with the probable result of saving more children, and yet there are a certain class of cases where the conjugate is so narrow that craniotomy has to be resorted to. In a certain number of these last cases, I believe the child's life might be saved by version. I have seen two cases where the mother had an extosis on the sympyses pubis, and craniotomy had to be performed when the head was the presenting part. After two such disastrous labors, one again became pregnant, and, when labor came on, a foot presented, with the result of a child born alive without mechanical interference. The other case was nearly identical with this one.

During the early history of the forceps, when it was little known, when it was roughly made and ignorantly used, it is not to be wondered at that its use was postponed until the last moment, even until the mother and child were nearly moribund, and if death subsequently ensued, the cause was naturally enough ascribed to the forceps, when, had it been earlier brought into requisition, such disastrous consequences need not have ensued. Absolute cleanliness, and the use of disinfectants, should always be strictly observed when these instruments are used; and when these conditions are present, they are, in the hands of a skilful operator, the means of saving the child-bearing woman an incalculable amount of suffering, saving hours of pain to the woman, and hours of anxiety to the friends. From 1790 to 1864, the average use of the forceps in the continental hospitals of Europe, according to Charpentier, was one in thirty, the minimum being Osiander, who used it only once in 291 labors, and the highest, Hohl, who used it once in every three labors. In Great Britain, from 1803 to 1863, John Clarke used it once in 3,878 labors, and Moore, of London, once in 291 labors. It is noticeable that Osiander, who used the forceps once in 291 confinements, did the work, upon which his statistics were based, between the years 1792 and 1822, and Hohl, who used it once in every three cases, from 1840 to 1857, showing a tendency, as time advanced, to use it more frequently. A plea for the use of the forceps somewhat earlier than formerly, may be found in Harper's tables, taken from Charpentier's work on obstetrics:

TABLE FROM HARPER.

| | Frequency of Application. | Fetal Mortality. | Maternal Mortality. | Duration Labor. |
|---|---|---|---|---|
| Collins . . . . . . . | 1–694 | 1–26 | 1–329 | 38 |
| Hardy . . . . . . . | 1–555 | 1–20 | 1–334 | 35 |
| Johnston . . . . . | 1–600 | 1–30 | 1–502 | 29 |
| Harper . . . . . . . | 1–260 | 1–47 | 1–1490 | 16 |

As there is at present a tendency among the profession in general to a much more frequent use of the forceps than formerly, I have tried to ascertain the number of times they are used in proportion to the number of cases attended, from a number of medical gentlemen in this city, with the following result: R. L. Banta, 1–10; A. H. Briggs, 1–6; F. W. Bartlett, 1–6; A. H. Crawford, 1–10; A. Dagenais, 1–12; E. T. Dorland, 1–6; C. C. Frederick, 1–8; Geo. E. Fell, 1–50; M. B. Folwell, 1–8; B. P. Hoyer, 1–4; M. Hartwig, 1–20; H. D. Ingraham, 1–11; Thos. Lothrop, 1–15; G. E. Mackey, 1–10; W. W. Potter, 1–4[1]; De Lancey Rochester, 1–23; Chas. E. Stockton, 1–20; E. Tobie, 1–11; W. H. Thornton, 1–15; C. C. Wyckoff, 1–10; J. J. Walsh, 1–2; W. D. Greene, 1–12—an average of once in about eleven and one-half cases. I regret being unable to obtain the death-rate also, whereby to make the statistics more complete. It is an undoubted fact that a great source of infant mortality, as shown by statistics, up to within eight or ten years has been the indiscriminate use of ergot. The physician had been taught that the use of forceps was wrong, except in extreme cases, but he had not been taught that the free use of ergot was also wrong, and therefore gave it *ad libitum.* After the uterus had been in tonic contraction for hours, it cannot be wondered at that a dead child was born, with or without the aid of forceps. I recognize the fact that, from not using the forceps often enough, we may err by going to the other extreme, and yet my own experience teaches me that using it once in every twelve or fifteen cases in a series of say 500 labors, is not too often. If no ergot whatever is given until labor is terminated, and the forceps used in delayed cases, I believe the infant mortality will decrease and the maternal mortality will not increase. It occurs to me we have struck a happy medium between two extremes. We should not use it as often as once in two or three cases, nor yet so seldom as once in 3,878 labors, neither should we employ the forceps without the use of germicides in the fullest sense of the word. In short, employ every means to potect mother and child; employ disinfectants, employ precaution, and above all, employ good judgment and common sense.

444 Elk Street.

1. In consultations chiefly.

